# Association between low serum prealbumin levels and carpal tunnel syndrome in maintenance hemodialysis patients

**DOI:** 10.1080/0886022X.2020.1811118

**Published:** 2020-09-11

**Authors:** Nguyen Huu Dung, Nguyen Duc Loc, Dao Bui Quy Quyen, Nguyen Minh Tuan, Pham Ngoc Huy Tuan, Do Quyet, Le Viet Thang

**Affiliations:** a Bach Mai Hospital, Hanoi, Vietnam; b An Sinh Hospital, Ho Chi Minh, Vietnam; c Cho Ray Hospital, Ho Chi Minh, Vietnam; d E Hospital, Ha Noi, Vietnam; e Trung Vuong Hospital, Ho Chi Minh, Vietnam; f Viet Nam Military Medical University, Hanoi, Vietnam; g Military Hospital 103, Hanoi, Vietnam

**Keywords:** Serum prealbumin, carpal tunnel syndrome, maintenance hemodialysis, low-flux dialysis reuse

## Abstract

**Aims:**

Carpal tunnel syndrome (CTS) and low serum prealbumin concentration are common in maintenance hemodialysis patients. In this study, we focused on the association between low serum prealbumin levels and carpal tunnel syndrome in maintenance hemodialysis (MHD) patients using low-flux dialysis reuse.

**Materials and methods:**

Serum prealbumin levels were assessed to determine the association between low serum prealbumin levels and CTS in 373 prevalent MHD patients (the mean age was 45 years old, hemodialysis duration was 46 months). The patients were divided into 2 groups: the CTS group with 44 patients and the non-CTS group with 329 patients.

**Results:**

The prevalence of CTS was 11.8%. Serum prealbumin showed a good prognostic value to predict CTS in MHD patients using low-flux dialysis reuse (the Area Under the Curve = 0.841, *p* < .001; cutoff value: 26.5 mg/dL with sensitivity = 72.7% and specificity = 79.9%).

**Conclusions:**

Serum prealbumin was a good prognostic biomarker of CTS in MHD patients using low-flux dialysis reuse.

## Introduction

Carpal tunnel syndrome (CTS) is the most common upper limb entrapment neuropathy, which occurs more frequently in the dominant hand and is related to underlying medical conditions or risk factors [1,2]. The most common symptoms of carpal tunnel syndrome are wrist pain, unpleasant tingling, hypesthesia on the distal end of the median nerve's sensory innervation and a reduction of grip strength. Long-term compression of the median nerve can lead to impaired functionality of the hand as well as hypotrophy or even atrophy of the thenar muscles [3,4]. The prevalence of carpal tunnel syndrome in hemodialysis patients correlates with the duration of hemodialysis treatment. CTS affects 30–50% patients who have been on hemodialysis for over 10 years and over 80% patients who have been on hemodialysis for over 30 years. Specific factors contribute to the development of carpal tunnel syndrome in patients on chronic hemodialysis, including β2-microglobulin (β2-M) fiber deposits and arteriovenous (AV) fistula [5–8]. Malnutrition-inflammation-atherosclerosis syndrome is a common cause of increased mortality in chronic kidney disease [9–11]. Prealbumin (transthyretin) is a hepatic secretory protein used to assess malnutrition in chronic disease patients, including maintenance hemodialysis patients [12–14]. Moreover, prealbumin has been implicated in systemic inflammatory processes in dialysis patients. The liver enhances prealbumin synthesis when the body experiences inflammation to increase the immune system to protect the body against inflammatory stimuli, especially patients with acute inflammation [15,16]. The association among malnutrition, chronic inflammation and CTS in hemodialysis patients has rarely been investigated. In this study, we hypothesize that a low serum prealbumin level is associated with CTS in maintenance hemodialysis patients using low-flux dialyzer.

## Methods

### Patients

There were 678 patients on prevalent hemodialysis (hemodialysis duration >3 months) who joined in our study at Hemodialysis Center, Bach Mai Hospital, Ha Noi, Viet Nam, as of March 2016. Of these, patients with acute illness, significant infection, malignancy, diagnosed CTS before chronic kidney disease, or used high-flux dialyzer were excluded. The remaining patients, including 373 prevalent hemodialysis patients, provided informed consent prior to participation in our study. The enrolled patients were treated with stable, regular hemodialysis using bicarbonate dialysate. Our dialysis program used a low-flux membrane (Polyflux 14 L) as a standard. Kt/V was calculated according to the formula of Daugirdas [17]. Each dialysis session was between 3.5 and 4.5 h to achieve the target total Kt/V of approximately 1.2 per session for thrice weekly treatments. Dialyzer was reused 6 times in all patients (the procedure is regulated by Vietnam's Ministry of Health) as followings. Reuse of dialyzer is performed by a professional, trained technician. After completing the dialysis session, the dialyzer is immediately transferred to the washing room. The dialyzer is cleaned by hand using RO water for 30 min. Next, the dialyzer is soaked and disinfected with 0.7% Peracetic acid solution and stored in a professional refrigerator at a temperature of 2–8 degrees C. Before use in the dialysis patient, the dialyzer is washed again with RO water for 30 min, and the lack of Peracetic acid in the dialyzer is confirmed using a Peracetic acid 2000 test strip.

Diabetes mellitus was identified according to a physician’s diagnosis, antidiabetic drug treatment, or 2 subsequent analyses demonstrating fasting blood glucose levels of >126 mg/dL or > 7.0 mmol/L. Hypertension was defined as the regular use of antihypertensive drugs to control blood pressure or at least 2 blood pressure measurements of >140/90 mmHg. Anemia was diagnosed as hemoglobin < 130 g/L for male and < 120 g/L for female. Patients with residual kidney function were diagnosed as described by Daugirdas [15], and lipid disorders were diagnosed based on the 2013 KDIGO (The Kidney Disease Improving Global Outcomes) Clinical Practice Guideline for Lipid Management in CKD (Chronic Kidney Disease) [18].

In addition, CTS diagnosis was made according to American Academy of Orthopedic Surgeons [19] with (1) signs or symptoms verified using nerve conduction examination; (2) clinical CTS diagnosis of nocturnal pain, numbness in the median nerve distribution, and a positive Tinel sign/Phalen sign; (3) prolonged sensory and/or motor latencies from the wrist to the digits innervated by the median nerve in the electrophysiological test; or (4) CTS requiring surgical release. We also defined hypoprealbuminemia as a serum prealbumin level of <30.0 mg/dL in maintenance hemodialysis (MHD) patients [20].

To identify an association between low serum prealbumin level and CTS, 373 patients were divided in to 2 groups: the CTS group (*n* = 44) and the non-CTS group (*n* = 329).

### Laboratory measurements

Blood was drawn immediately before the start of a dialysis session in a non-fasting state to measure hemoglobin, hematocrit, serum albumin, creatinine, blood urea nitrogen, lipid components, high sensitive C-reactive protein (hs-CRP) and β2-M using routine laboratory methods, and this process is performed once a month as routine clinical care as performed in most dialysis facilities in Viet Nam. HBsAg and anti-HCV antibody status was assessed by serological testing in all MHD patients. Serum prealbumin concentration was measured using quantitative electrochemiluminescence *method* (*ECLIA*) at the time of enrollment.

### Statistical analyses

All the normally distributed continuous data are presented as the mean and standard deviation and were analyzed by Student’s *t*-test. All the skewed distributions are presented as the median (25–75 percentile) and were analyzed by the Mann Whitney U test. Categorical data were presented by frequency with percentage and were analyzed using Chi-square test. Multivariable adjusted regression analysis was performed to identify the predictors of CTS. Receiver operating characteristic (ROC) curves with the area under the curve (AUC) was calculated to predict CTS from all patients. Statistical analysis was performed using Statistical Package for Social Science (SPSS) version 20.0 (Chicago, IL, USA). A *p*-value <.05 was considered significant.

## Results

Table 1 shows that mean age is 45.6 years old, and 57.4% of the patients are males. In total, 79.1% of patients exhibited no residual kidney function, and diabetic mellitus was noted in 9.1% patients. In total, 30.8% of the patients experienced shoulder pain. Specially, there were 11.8% patients with CTS and 39.4% patients with low serum prealbumin.

**Table 1.** Clinical characteristics and laboratory parameters of the studied patients (*n* = 373).

Clinical characteristics and laboratory parameters	Mean ± SD/Median (IQR)	*n*, %
Age (years)	45.61 ± 14.42	N/A
Number of male, *n* (%)	N/A	214 (57.4)
Duration of hemodialysis (months)	46 (34–56)	N/A
No residual kidney function, *n* (%)	N/A	295 (79.1)
Diabetes, *n* (%)	N/A	34 (9.1)
Hypertension, *n* (%)	N/A	272 (72.9)
Anemia, *n* (%)	N/A	315 (84.5)
Shoulder pain, *n* (%)	N/A	115 (30.8)
Hepatitis infection, *n* (%)		
HBV	N/A	29 (7.8)
HCV	N/A	110 (29.5)
HBV + HCV	N/A	16 (4.3)
BMI (kg/m^2^)	19.13 ± 2.31	N/A
Blood urea (mmol/l)	29.5 ± 6.83	N/A
Serum creatinine (µmol/l)	836 (678–986)	N/A
Albumin (g/L)	38.66 ± 3.48	N/A
Hemoglobin (g/L)	103.02 ± 18.55	N/A
Cholesterol (mmol/L)	4.04 (3.38–5.21)	N/A
Triglyceride (mmol/L)	1.56 (1.07–2.35)	N/A
LDL-C (mmol/L)	2.2 (1.63–2.98)	N/A
Hs-CRP (mg/L)	0.4 (0.1–0.7)	N/A
β2-M (mg/L)	66.8 (48.55–79.95)	N/A
Prealbumin		
Median (IQR)	34 (26–42)	N/A
<30 mg/dL, *n* (%)	N/A	147 (39.4)
Carpal tunnel syndrome, *n* (%)	N/A	44 (11.8)

HBV: Hepatitis B Virus; HCV: Hepatitis C Virus; BMI: Body Mass Index; LDL-C: Low-density Lipoprotein-Cholesterol; hs-CRP: high-sensitivity C-Reactive Protein; β2-M: Beta2-Microglobulin; IQR: Interquartile range.

Comparisons of the clinical characteristics and laboratory parameters between the CTS and without CTS groups revealed that the mean age, female rate, median duration of hemodialysis, ratio of shoulder pain, prevalence of HBV, HCV infection, median hs-CRP level, median β2-M concentration and ratio of patients with low serum prealbumin in the CTS group were significantly increased compared with the without CTS group (*p* < .05, .001). In contrast, mean serum albumin and median serum prealbumin levels in the CTS group were significantly reduced compared with the group without CTS (*p* < .05).

Serum prealbumin concentration exhibits improved predictive value for CTS than serum albumin in MHD patients.

## Discussion

In our study, the ratio of CTS was 11.8% (44/373) in MHD patients with a median duration of hemodialysis of 46 months. The result was similar to previous studies. Kopec J *et al.* [21] reported in their study of 386 MHD patients that CTS was diagnosed in 40 patients (10.4%) based on signs and physical symptoms as well as nerve conduction. Huang WH *et al* reported 76 CTS patients in a study of 866 MHD patients (8.8%) [22]. However, some authors reported an increased ratio of CTS in MDH patients compared with our study, i.e. 27.5% (11/40 MHD patients) [23] and 30.5% (18/59 MHD patients) [24]. The incidence of CTS in different studies is related to different patient characteristics, and the frequency of occurrence of CTS risk factors differed in the studies. In our study, old age, female sex, long duration of dialysis, hepatitis virus infection, serum hypoalbuminia, elevated serum CRP, high serum β2-M level and low serum prealbumin were related to the occurrence of CTS in MHD patients (Table 2). However, in multivariate logistic analyses, we found that female sex, long duration of hemodialysis, shoulder pain and prealbumin levels are independently related to the appearance of CTS (Table 3). Mitake T *et al.* confirmed that sex difference is a risk factor of CTS. These researchers suggested that CTS especially in male patients might be reduced by early interventions for diabetes mellitus [25]. A previous cohort study showed the increased occurrence of CTS in long-term HD patients [26]. Kopec ´ J *et al.* [21] reported a positive correlation between HCV infection and CTS in MHD patients. They reported that the longer patients are dialyzed, the greater their probabilities of HCV infection. In our study, patients with CTS exhibited an increased frequency of HCV infection (68.4% in CTS group versus 24.3% in non-CTS group, *p* < .001). The use of a long low-flux membrane will increase serum β2-M concentrations in MHD patients, which is a risk factor of CTS. The result is clearly demonstrated in Table 2. Specifically, serum β2-M levels were 82.45 mg/L in CTS group and 64.1 mg/L in patients without CTS. Malnutrition in MHD patients is a well-established condition and often co-exists with inflammation. Approximately 50 to 75% of MHD patients exhibit symptoms of malnutrition-inflammation complex syndrome depending on the diagnostic tool used [27]. The relationship between inflammation and CTS as well as malnutrition and CTS in MDH patients was confirmed in some previous reports [28,29]. This relationship was once again confirmed in our study. In our group of CTS patients, the prevalence of hepatitis virus infection and hs-CRP concentration were increased and the serum albumin concentration was significantly reduced compared with the non-CTS patient group (Table 2). In our study, the ratio of diabetes in CTS patients was increased compared with the non-CTS group, *p* = .095 (Table 2). Our research results are not consistent with previous studies [30,31]. Diabetes mellitus (DM) has been proposed as a risk factor for carpal tunnel syndrome, although the exact cause and pathogenesis of CTS remain unclear. We believe that the number of diabetic patients in this study (only 9.1%) does not affect our research results. In addition to diabetes mellitus, this type of membrane also affects the incidence of CTS in dialysis patients [21,32]. The use of a high-flux membrane, which eliminates larger molecular weight solutes, including β2-M, is directly related to increasing ratios of CTS in maintenance hemodialysis patients.

**Table 2.** Comparison of some clinical characteristics and laboratory parameters between MHD patients with CTS and without CTS.

Clinical characteristics and laboratory parameters	CTS, (*n* = 44)	Non-CTS, (*n* = 329)	*p*
Age (years), Mean ± SD	52.16 ± 12.6	44.74 ± 14.44	*<.001*
Number of females, *n* (%)	30 (68.2)	129 (39.2)	*<.001*
Duration of hemodialysis (months), Median (IQR)	114 (89.5–140)	40 (24–70.5)	*<.001*
No residual kidney function, *n* (%)	39 (88.6)	256 (77.8)	.097
Diabetes, *n* (%)	7 (15.9)	27 (8.2)	.095
Hypertension, *n* (%)	29 (65.9)	243 (73.9)	*.265*
Anemia, *n* (%)	36 (81.8)	279 (84.8)	.608
Shoulder pain, *n* (%)	42 (95.5)	73 (22.2)	*<.001*
Hepatitis infection, *n* (%)			
HBV	2 (4.5)	27 (8.2)	*<.001*
HCV	30 (68.2)	80 (24.3)	
HBV + HCV	4 (9.1)	12 (3.6)	
BMI (kg/m^2^), Mean ± SD	19.63 ± 2.93	19.07 ± 2.22	.225
Blood urea (mmol/l), Mean ± SD	33.26 ± 6.44	28.99 ± 6.73	*<.001*
Serum creatinine (µmol/l), Median (IQR)	789 (659.5–933)	846 (678–987)	.206
Albumin (g/L) , Mean ± SD	37.84 ± 2.22	38.77 ± 3.60	*.02*
Hemoglobin (g/L), Mean ± SD	105.39 ± 18.62	102.71 ± 18.55	.369
Cholesterol (mmol/L), Median (IQR)	4.49 (3.49–5.31)	4.01 (3.37–5.14)	.109
Triglyceride (mmol/L), Median (IQR)	1.91 (1.06–2.52)	1.53 (1.07–2.31)	.237
LDL-C (mmol/L), Median (IQR)	2.41 (1.54–3.29)	2.18 (1.65–2.91)	.309
Hs-CRP (mg/L), Median (IQR)	0.5 (0.22–0.95)	0.4 (0.1–0.6)	*.016*
β2-M (mg/L), Median (IQR)	82.45 (72.1–92.6)	64.1 (46.1–77.3)	*<.001*
Prealbumin			
Median (IQR)	21 (16–27.75)	36 (28–43)	*<.001*
<30 mg/dL, *n* (%)	36 (81.8)	111 (33.7)	*<.001*

HBV: Hepatitis B Virus; HCV: Hepatitis C Virus; BMI: Body Mass Index; LDL-C: Low-density Lipoprotein-Cholesterol; hs-CRP: high-sensitivity C-reactive Protein; β2-M: Beta2-Microglobulin; IQR: Interquartile range.

Italic values showed the statistical significance of the comparison (*p* < 0.05).

**Table 3.** Multivariate logistic regression analysis between CTS and clinical variables in MHD patients.

Variable	Adjusted hazard ratio	95% Cl	*p*
Female sex	5.918	1.844–18.989	*.003*
Duration of hemodialysis (months)	1.04	1.023–1.058	*<.001*
Shoulder pain (Yes)	38.024	7.286–198.428	*<.001*
Hemoglobin <100 (g/L)	0.387	0.123–1.132	.083
Prealbumin (mg/dL)	0.906	0.854–0.962	*.001*

Female sex, long duration of hemodialysis, shoulder pain, and prealbumin levels are independent predictors of CTS (*p* < .005).

Italic values showed the statistical significance of the comparison (*p* < 0.05).

**Figure 1.**
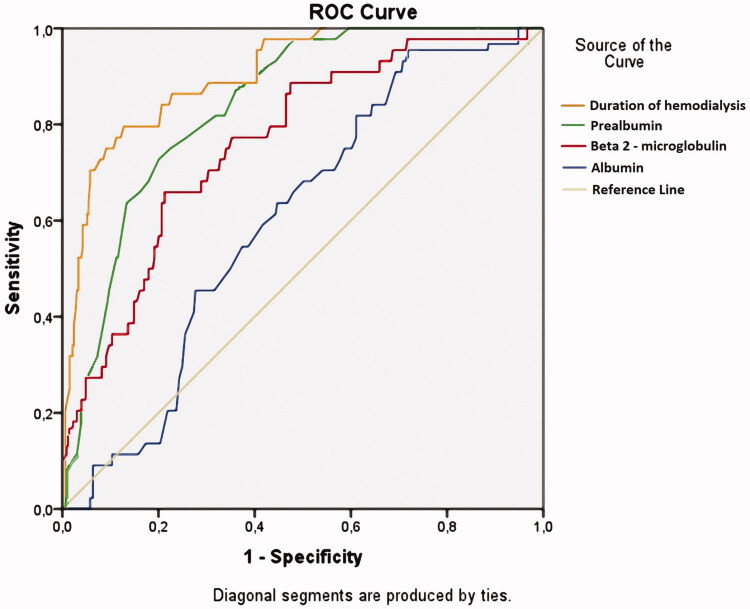
Receiver operating characteristics (ROC) curves of duration of hemodialysis, serum β2-M, and prealbumin for prediction of CTS of MHD patients. Duration of hemodialysis: AUC = 0.903; *p* < .001; Cutoff value: 84.5 months, Se= 79.5%, Sp= 87.2%. Serum prealbumin: AUC = 0.841; *p* < .001; Cutoff value: 26.5 mg/dL, Se= 72.7%, Sp= 79.9%. β2-M: AUC = 0.762; *p* < .001; Cutoff value: 78.85 mg/L, Se= 65.9%, Sp = 78.7%. Serum albumin: AUC = 0.604; *p* = .026; Cutoff value: 40.75 g/L, Se= 95.5%, Sp = 28.3%.

Protein-energy malnutrition in dialysis patients is frequently noted, multifactorial, and typically begins well before the start of hemodialysis. Protein-energy malnutrition results from an imbalance between the contributions and the needs of the organism, resulting in tissue losses with deleterious functional consequences associated with a high morbidity and with an unfavorable prognosis, and this notion was confirmed in our previous studies [33]. Prealbumin is an acute phase protein, and serum prealbumin levels are regarded as a reliable indicator for evaluating nutritional states and the effect of nutritional intervention in MDH patients. In addition, prealbumin levels are protein energy wasting diagnostic criteria [20]. Prealbumin and albumin are important in nutritional assessment as well as predictors of mortality in MHD patients [13]. In this study, we aimed to determine the independent association of serum prealbumin with CTS in MHD patients, and we built ROC curves of duration of hemodialysis, serum β2-M level, and prealbumin level to predict CTS in MHD patients. Our results reconfirmed that duration of hemodialysis, serum β2-M levels, and serum albumin levels exhibit predictive values in CTS. In particular, serum prealbumin levels exhibited predictive values greater than those of β2-M and albumin but less than hemodialysis duration (Figure 1). The results confirmed the association between low serum prealbumin levels (<30.0 mg/dL) and carpal tunnel syndrome in maintenance hemodialysis patients who used low-flux membranes and reused dialyzer.

Although our research results have addressed the research objectives, this study has some limitations. First, the study was adopted as a cross-sectional study design. Second, our study did not clearly assess other risk factors of CTS, such as arteriovenous fistula, high fat/muscle ratio, physical activity and lead exposure in MHD patients. Finally, this is single center study, so other studies should assess the relation between CTS and other diseases, such as diabetes, as well as the type of dialyzer membrane.

## Conclusion

In conclusion, a low serum prealbumin concentration was a good predictor of CTS in maintenance hemodialysis patients.
